# Propofol Infusion Syndrome in the Postoperative Period of a Kidney Transplant

**DOI:** 10.1155/2019/7498373

**Published:** 2019-09-25

**Authors:** Edgar Dehesa-López, Sergio Saul Irizar-Santana, Rolando Claure-Del Granado, Rafael Valdez-Ortiz

**Affiliations:** ^1^Department of Internal Medicine and Nephrology, Hospital Civil de Culiacán, Culiacán, Mexico; ^2^Centro de Investigación y Docencia en Ciencias de la Salud, Universidad Autónoma de Sinaloa, Culiacán, Mexico; ^3^Instituto Mexicano del Seguro Social (IMSS), Mexico; ^4^Division of Nephrology, Hospital Obrero #2 – C.N.S., Cochabamba, Bolivia; ^5^Department of Nephrology, Hospital General de México, Mexico City, Mexico

## Abstract

Sedation during medical procedures poses a risk to any patient, and the use of specific anesthetic agents should be carefully considered to avoid adverse outcomes. We report on a patient with propofol infusion syndrome diagnosed during the post-operative period of a renal transplant. A 58-year-old female on chronic hemodialysis due to end stage kidney disease secondary to microscopic polyangiitis underwent kidney transplant from a deceased donor. Anesthetic induction was performed with fentanyl, propofol, and cisatracurium, and maintained with continuous propofol infusion. In the recovery room, the patient developed somnolence, tachypnea, and thoracoabdominal dissociation secondary to residual neuromuscular block. An arterial-blood gas test indicated acidemia, high pCO_2_, low HCO_3_, and mildly increased serum lactate. The patient remained hemodynamically stable, on volume-controlled ventilation, with sedation by continuous propofol infusion. Blood gas tests revealed persistent acidemia without tissue hypoperfusion. Doppler ultrasound of the renal graft reported adequate blood flow and serum triglycerides were elevated. A diagnosis of propofol infusion syndrome was made, and infusion ceased. A decrease in serum lactate levels was observed, with normalization 4 h later. This case highlights the importance of considering adverse effects of anesthetic agents as the cause of post-operative complications when prolonged sedation is required.

## 1. Introduction

Renal transplantation is currently the best therapeutic option for treating end-stage kidney disease (ESKD) [1, 2]. Current pre-transplant evaluation of the anesthetic, medical, surgical, and immunological risks of the renal recipient makes this a safe procedure, with a low complication rate [3–5]. Propofol is a powerful general anesthetic agent with a short half-life, and characterized by its immediate action and rapidly reversible sedation, which make it popular for sedation during medical or surgical procedures and for maintained sedation in ICUs [6, 7]. Propofol infusion syndrome (PRIS) is a rare, potentially fatal condition first described in children in the 1990s, and later reported in adults. It is generally associated with high doses or prolonged use of propofol infusion, and characterized by metabolic acidosis, rhabdomyolysis of skeletal and cardiac muscle, arrhythmias (bradycardia, atrial fibrillation, ventricular and supraventricular tachycardia, bundle branch block, and asystole), myocardial failure, renal failure, and hepatomegaly [8]. We present a case of severe lactic acidosis, secondary to PRIS, in the post-operative period of a kidney transplant.

## 2. Case Presentation

**Figure 1:**
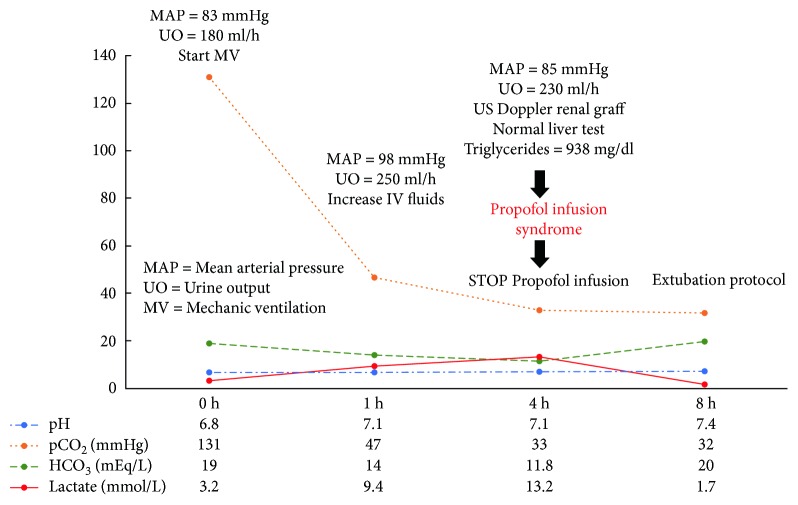
Evolution of gasometric parameters during treatment of patient.

**Figure 2:**
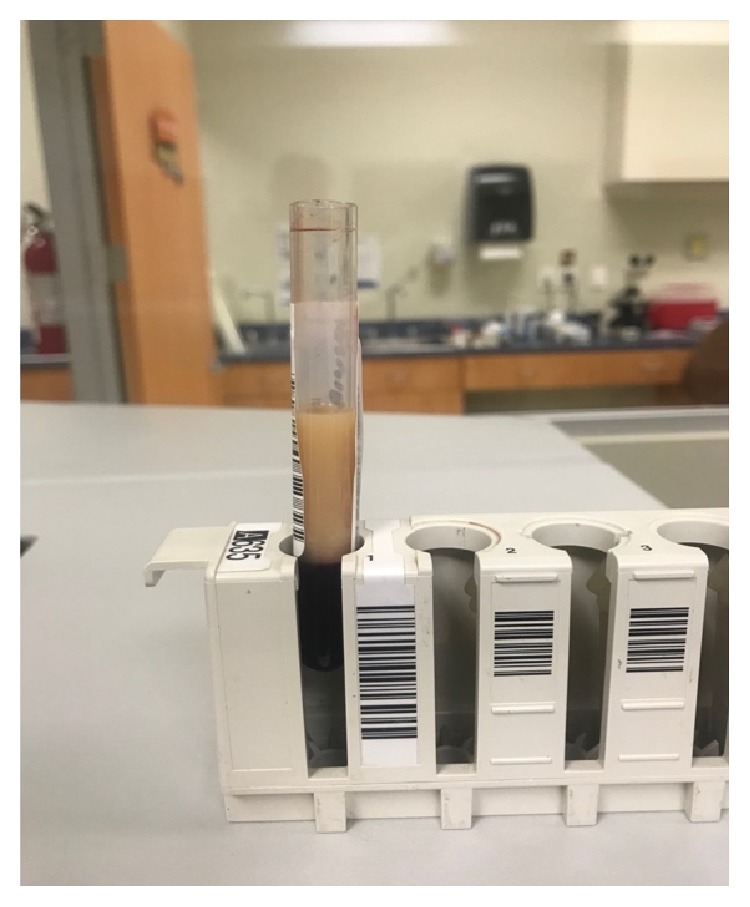
Image shows the patient's lipemic serum.

A 58-year-old female was diagnosed with ESKD secondary to microscopic polyangiitis, refractory to initial treatment with methylprednisolone, intravenous cyclophosphamide, and plasmapheresis. She remained on chronic, intermittent hemodialysis 3 times a week for 16 months and had diuresis (800 ml/24 h), without any clinical or biochemical activity of microscopic polyangiitis in the past 6 months. She successfully completed the protocol for a kidney transplant from a deceased donor, which was performed in August of 2018. Crossmatch test was negative, blood type was A+ (donor 26-year-old female, blood type O+), and the induction immunosuppression regimen used was methylprednisolone (1000 mg, IV) and basiliximab (20 mg, IV). The surgical procedure was performed under total general intravenous anesthesia, induced with fentanyl (3 *µ*/kg), propofol (1.5 mg/kg), and cisatracurium (100 *µ*/kg). Anesthesia was maintained with continuous infusion of propofol (100–200 *µ*/Kg/min). Two arterial anastomoses, one venous and one ureterovesical, were made without complications. Adequate perfusion of the graft was corroborated, and 200 ml of spontaneous diuresis was observed by the end of the surgical procedure. The patient remained hemodynamically stable during surgery, which lasted 3 h, with a cold ischemia time of 10 h and surgical bleeding less than 500 ml. In the recovery room, the patient developed somnolence, tachypnea, and thoracoabdominal dissociation secondary to residual neuromuscular block. An arterial-blood gas test revealed a pH of 6.8, pCO_2_ 131 mmHg, pO_2_ 212 mmHg, HCO_3_ 19 mmol/L, and lactate 3.2 mmol/L. The patient was intubated and transferred to the intensive care unit (ICU) for mechanical ventilation support. In the ICU, the patient remained on volume-controlled ventilation, was hemodynamically stable, and remained sedated with continuous propofol infusion at 0.5 mg/kg/h. A venous-blood gas test performed 1 h later showed that pH increased to 7.10, pCO_2_ 47 mmHg, pO_2_ 59 mmHg, HCO_3_ 14 mmol/L, and increased lactate levels (9.4 mmol/L) (Figure 1). Common causes of type A and B lactic acidosis were ruled out due to a lack of arterial hypotension and tissue hypoperfusion, presence of spontaneous renal graft diuresis, normal oxygen saturation, and normal liver tests. Three hours after increasing intravenous fluids, a new venous-blood gas test showed persistent acidemia with a pH of 7.16, pCO_2_ 33 mmHg, pO_2_ 60 mmHg, HCO 11.8 mmol/L, and increased lactate levels (13.2 mmol/L), despite maintaining hemodynamic stability, with MAP >65 mmHg without vasopressor treatment, urinary output 200–300 ml/h, and being afebrile without any clinical data demonstrating tissue hypoperfusion (Figure 1). No auricular or ventricular arrhythmia was documented, and her serum level of creatine phosphokinase was normal (143 U/L). Doppler ultrasound of the renal graft reported adequate blood flow at the principal and polar renal arteries. Once the main causes of lactic acidosis were ruled out, we reviewed less frequent causes of unexplained lactic acidosis and found that propofol infusion syndrome (PRIS) could be the cause in our patient. We proceeded to measure serum triglyceride levels, which were 964 mg/dl (Figure 2), and the patient was diagnosed with PRIS. Propofol infusion was stopped, and correction of metabolic acidosis and a progressive decrease in serum lactate levels were observed, with normalization 4 h later (pH 7.4, pCO_2_ 32 mmHg, HCO_3_ 20 mmol/L, and lactate 1.7 mmol/L) (Figure 1). The patient was extubated without complications and transferred to the hospital ward. Progressive decrease and normalization of serum triglyceride and creatinine levels were documented, and she was discharged on postoperative day 4 with no other complications.

## 3. Discussion

The first cases of a rare, but potentially lethal complication associated with the use of propofol infusion were reported in pediatric patients in 1990 [9]. This complication was characterized by severe lactic acidosis, rhabdomyolysis, and increased lipemia, with cardiac, hepatic, and renal dysfunction. These pediatric cases were then followed by an increasing number of reported cases in adults, especially in those treated with high doses (>5 mg/kg/h) or prolonged infusions (>48 h) of propofol [10].

The incidence rate of PRIS is difficult to assess due to the use of different diagnostic criteria. Roberts et al. prospectively studied 1,017 ICU patients at 11 academic medical centers treated with propofol infusion for at least 24 h, reporting an incidence of 1.1% [10]. Hemphill et al. recently conducted a narrative review of all cases of PRIS published between 1989 and 2018, documenting a total of 168 cases, of which 44 were in the pediatric population and 124 in the adult population [11]. Since there is variability in the diagnostic criteria used, the authors proposed a definition for PRIS, which is a syndrome that occurs in critically ill patients receiving either high doses (>5 mg/kg/h) or long duration (>48 h) propofol infusions, with one or more of otherwise unexplained symptoms including metabolic acidosis, rhabdomyolysis, electrocardiographic changes with or without acute kidney injury, hyperkalemia, lipidemia, cardiac failure, fever, elevated liver enzymes, or increased lactate levels.

Hemphill et al. also proposed to classify clinical features of PRIS into primary and secondary. Primary features include metabolic acidosis, electrocardiographic changes, and rhabdomyolysis, and secondary features include acute kidney injury, hyperkalemia, lipidemia, cardiac failure, fever, elevated liver enzymes, and increased lactate levels [11]. According to this classification, the diagnosis of PRIS in our patient was based on the presence of 1 primary clinical feature (metabolic acidosis) and 2 secondary features (raised serum lactate levels, and lipidemia).

The primary features most frequently reported by the authors were metabolic acidosis (77%), electrocardiographic changes (62.8%), and rhabdomyolysis (62%). Secondary features most frequently observed were acute kidney injury (50.4%), hyperkalemia (33.6%), raised lactate serum levels (31%), arterial hypotension (31%), cardiac failure (25%), and lipidemia (22.1%) [11]. Multisystem involvement was a frequent finding, with involvement of 3 organs in 29.2%, 4 organs in 23.9%, 2 organs in 20.4%, and 6 organs in 5.3% of cases, with only 15.9% of cases presenting with the involvement of a single organ [11].

From the first reported cases, a wide variety of risk factors for developing PRIS have been identified, including cumulative propofol dose [12, 13], infusion duration [13], sepsis [14], steroids [15], vasopressors [16], fasting [15], critical illness [10], rich fat and low carbohydrate diet [14], inborn errors in mitochondrial fatty-acid oxidation [17], and cranioencephalic trauma [12]. The main risk factors consistently associated with PRIS are accumulated dose and duration of propofol infusion [11, 13]. However, it is important to emphasize that cases of PRIS with multisystem compromise have been reported with low doses of propofol [18]. Krajcova et al. reviewed 153 cases of PRIS and were able to differentiate features dependent on dosage from those related to infusion duration [13]. Features of PRIS dependent on dosage were heart failure, metabolic acidosis, fever, and hypotension. Features dependent on infusion duration were arrhythmias and electrocardiographic changes. Features dependent on high dosage and prolonged duration were rhabdomyolysis and hypertriglyceridemia [13]. Finally, the idiosyncratic features of PRIS (independent of dose and infusion duration) most frequently observed were hepatomegaly and acute kidney injury [13]. In our case, the patient presented a mild form of PRIS due to early diagnosis and immediate discontinuation of propofol infusion.

In our case, the risk factors that likely contributed to the development of PRIS were severe respiratory acidosis during post-anesthetic recovery, use of high doses of methylprednisolone during the induction of immunosuppression, and ESKD. The latter may have caused accumulation of propofol, despite using the recommended doses and a short infusion time. Ickx et al. documented that the pharmacokinetics and pharmacodynamics of propofol are not affected during the induction and maintenance of general anesthesia in patients with ESKD, and that the kidneys do not contribute significantly to extra-hepatic clearance [19]. However, Fodale et al. more recently showed that kidneys have an important role in intrinsic clearance of propofol, contributing up to 27% of total body clearance [8]. An explanation for these contradictory results is use of different pharmacokinetic models to determine total body clearance of propofol [20].

The pathophysiology of PRIS is complex and multifactorial. It is characterized by injury and cell death secondary to imbalance between energy supply and demand within the cells. Propofol interferes with mitochondrial fatty-acid oxidation by inhibiting the enzymatic activity of palmitoyl-transferase I, which causes an energy deficit within cells, and accumulation of free fatty acids in the serum and various organs [21, 22]. This energy deficit mainly affects skeletal and cardiac muscle cells, causing rhabdomyolysis and myocardial dysfunction, respectively [15]. Rhabdomyolysis leads to the release and elevation of intracellular products such as myoglobin, creatinine phosphokinase, potassium, and lactic acid, which can trigger or aggravate kidney damage and acidosis. At the cardiovascular level, accumulation of free fatty acids has been associated with cardiac arrhythmias [15]. Propofol inhibits *β*-adrenergic receptors and blocks cardiac calcium channels, which results in a shock state that can evolve to refractory cardiogenic shock, requiring intravenous catecholamines, which can also trigger or aggravate kidney damage and acidosis [15, 23, 24]. It is important to emphasize that these features can often be explained by the patient's critical illness (sepsis, septic shock, traumatic brain injury, etc.), which can mask PRIS and delay its diagnosis.

The prognosis of PRIS is variable and depends of severity and degree of systemic involvement. Hemphill et al. documented a mortality rate of 48% in adult patients and 52% in pediatric patients with the main factors related to mortality being propofol infusion rate, accumulated dose, and infusion duration [11]. Clinical features associated with mortality in adult patients were electrocardiographic changes, arterial hypotension, hyperkalemia, and cranioencephalic trauma. Systemic involvement of multiple organs was also associated with higher mortality in adult patients [11]. Our patient did not present any of the clinical features or dose/duration criteria associated with propofol-induced mortality.

Treatment of PRIS includes immediate suspension of propofol infusion, general support measures, and cardiopulmonary and renal support, if necessary [25]. There is no specific treatment, so early diagnosis is crucial for successful treatment. Awareness of clinical profiles and increased monitoring of patients receiving propofol infusion constitutes the best measures for an early diagnosis. Specific recommendations to reduce the incidence of PRIS are: avoid infusions >5 mg/kg/h and for >48 h. In addition, carbohydrates should be provided to prevent lipemia. It is important to monitor blood pH, lactate levels, and creatine phosphokinase in patients receiving high doses or prolonged infusions of propofol, which cannot be avoided [11, 15, 26].

## 4. Conclusions

PRIS is a rare but potentially lethal complication that usually occurs in patients receiving propofol infusions at either high doses (>5 mg/kg/h) or for long durations (>48 h). Clinical features and systemic involvement are variable; therefore, awareness of the clinical entity and high level of suspicion are the best measures for early diagnosis. PRIS should be considered as a differential diagnosis in patients on propofol infusion who develop unexplained severe lactic acidosis.
